# Inhibition of WIP1 phosphatase sensitizes breast cancer cells to genotoxic stress and to MDM2 antagonist nutlin-3

**DOI:** 10.18632/oncotarget.7363

**Published:** 2016-02-13

**Authors:** Sona Pechackova, Kamila Burdova, Jan Benada, Petra Kleiblova, Gabriela Jenikova, Libor Macurek

**Affiliations:** ^1^ Department of Cancer Cell Biology, Institute of Molecular Genetics of the ASCR, CZ-14220 Prague, Czech Republic; ^2^ Institute of Biochemistry and Experimental Oncology, Charles University in Prague, CZ-12853 Prague, Czech Republic

**Keywords:** WIP1 inhibitor, p53, checkpoint, nutlin-3, breast cancer

## Abstract

PP2C family serine/threonine phosphatase WIP1 acts as a negative regulator of the tumor suppressor p53 and is implicated in silencing of cellular responses to genotoxic stress. Chromosomal locus 17q23 carrying the *PPM1D* (coding for WIP1) is commonly amplified in breast carcinomas and WIP1 was proposed as potential pharmacological target. Here we employed a cellular model with knocked out *PPM1D* to validate the specificity and efficiency of GSK2830371, novel small molecule inhibitor of WIP1. We have found that GSK2830371 increased activation of the DNA damage response pathway to a comparable level as the loss of *PPM1D*. In addition, GSK2830371 did not affect proliferation of cells lacking *PPM1D* but significantly supressed proliferation of breast cancer cells with amplified *PPM1D*. Over time cells treated with GSK2830371 accumulated in G1 and G2 phases of the cell cycle in a p21-dependent manner and were prone to induction of senescence by a low dose of MDM2 antagonist nutlin-3. In addition, combined treatment with GSK2830371 and doxorubicin or nutlin-3 potentiated cell death through a strong induction of p53 pathway and activation of caspase 9. We conclude that efficient inhibition of WIP1 by GSK2830371 sensitizes breast cancer cells with amplified *PPM1D* and wild type p53 to chemotherapy.

## INTRODUCTION

Cells exposed to genotoxic stress protect their genome integrity by activation of a conserved DNA damage response pathway that orchestrates DNA repair and represents an intrinsic barrier preventing genome instability and tumorigenesis [1, 2]. A core component of this pathway is the tumor suppressor p53 that controls cell fate decisions. Depending on the amplitude and duration of its activation, p53 promotes temporary cell cycle arrest (checkpoint), permanent withdrawal from the cell cycle (senescence) or programmed cell death (apoptosis) [3–5]. Under basal conditions, function of the p53 is suppressed by an E3 ubiquitin ligase MDM2 and its enzymatically inactive homologue MDMX that control p53 stability and transcriptional activity, respectively [6, 7]. Genotoxic stress triggers activation of ATM/ATR, Chk1/Chk2 and other kinases that extensively phosphorylate the N-terminal domain of p53, MDM2 and MDMX allowing stabilization of the p53 and promoting expression of its target genes [8–11]. One of the p53 target genes is *PPM1D* that codes for a Protein phosphatase 2C isoform delta (hereafter referred to as WIP1) [12]. Expression of WIP1 is induced by genotoxic stress and forming a negative feedback loop, WIP1 efficiently inhibits the p53 pathway by a direct dephosphorylation of p53 at Ser15 and also by dephosphorylation of its negative regulators MDM2 and MDMX [13–16]. By inactivating the p53 pathway, WIP1 promotes recovery from the G2 checkpoint [17, 18]. Moreover, WIP1 dephosphorylates other proteins including ATM, Chk1, Chk2, p38 and γH2AX which contributes to the termination of the DNA damage response [19–24]. In addition, WIP1 was reported to prevent premature senescence in various cell types and tissue compartments [21, 25, 26].

Chromosomal locus 17q23 carrying the *PPM1D* gene is commonly amplified in various human tumors including breast, ovarian and gastric cancer, neuroblastoma and lung adenocarcinoma [27–34]. In particular, amplification of the *PPM1D* occurs in approximately 10 % of breast tumors, typically those that retain wild type p53 [31, 35, 36]. In addition, about one third of breast tumors with amplified *PPM1D* locus also contain amplification of the *ERBB2/HER2* oncogene suggesting that both genes may jointly promote tumor development [36]. Indeed, MMTV-driven overexpression of *Ppm1d* potentiated *Erbb2*-induced breast tumor development in mice [37]. Comparably less common than *PPM1D* amplifications are rare nonsense mutations in the exon 6 of *PPM1D* that result in expression of abnormally stable WIP1 and promote development of breast and ovary cancer [38–40].

Reactivation of the p53 function by various MDM2 or MDMX antagonists and other small molecule p53 activators has been proposed as promising strategy for treatment of cancers with the wild-type p53 [41–45]. Nutlin-3 is a potent and selective antagonist of the interaction between MDM2 and p53 (IC50 of 90 nM) [46]. Treatment with nutlin-3 activates the p53 pathway and depending on the dose induces cell cycle arrest or cell death [46]. RG7388, an orally available analogue of nutlin-3, efficiently suppressed tumor growth *in vivo* [47]. Clinical trials are currently ongoing to prove clinical efficacy of MDM2 antagonists in cancer therapy. Reactivation of p53 pathway can be also achieved by inhibition of WIP1 and indeed WIP1 was proposed a potential pharmacological target in cancer therapy [21, 48]. Loss of *Ppm1d* dramatically delayed the development of Erbb2-induced breast cancer, MYC-induced lymphoma and APC^min^-induced intestinal tumors in mice [49–52]. In addition, depletion of WIP1 using RNA interference has been shown to efficiently suppress growth of various human cancer cells [30, 53–55]. However, translation of these observations into clinics is challenging due to the lack of suitable WIP1 inhibitors with sufficient specificity and favourable pharmacokinetic properties. Cyclic phosphopeptides that mimic substrates of WIP1 can block its phosphatase activity *in vitro*, but their efficiency in cells still remains to be addressed [56, 57]. A high-throughput screening identified a small molecule CCT007093 that inhibited WIP1 *in vitro* (IC50 = 8.4 μM) and eradicated WIP1 overexpressing tumor cells [58]. However, the specificity of CCT007093 towards WIP1 may be low in cells [59]. Small molecules SPI-001 and its analogue SL-176 inhibited WIP1 *in vitro* (IC50 = 86.9 nM and 110 nM and, respectively) and supressed growth of cells with the C-terminally truncated or overexpressed WIP1 but their efficiency at organismal level still needs to be tested [60–62]. Novel orally available inhibitor of WIP1 phosphatase GSK2830371 has recently been shown to selectively inhibit WIP1 *in vitro* (IC50 = 6 nM) and to efficiently suppress growth of a subset of hematopoietic tumor cell lines and neuroblastoma cells with overexpression of WIP1 [63, 64].

Here we aimed to validate the specificity and efficiency of the commercially available WIP1 inhibitors in blocking proliferation of the breast cancer cells. We have found that GSK2830371 suppressed growth of breast cancer cells with amplified *PPM1D* gene in a p53-dependent manner which is in good agreement with previous RNAi-based studies. In addition, we have found that inhibition of WIP1 is not sufficient to induce cell death in cancer cells but rather slows down proliferation by extending G1 and G2 phases of the cell cycle. However, breast cancer cells treated with WIP1 inhibitor are more sensitive to DNA damage-inducing chemotherapy and to MDM2 antagonist nutlin-3. Combined treatment with these drugs triggers senescence or programmed cell death and can efficiently eradicate p53 positive breast cancer cells. Our data validate GSK2830371 as potent and selective inhibitor of WIP1 that sensitizes breast cancer cells to chemotherapy.

## RESULTS

### WIP1 inhibition impairs proliferation of breast cancer cells with amplified PPM1D and wt-p53

**Figure 1:**
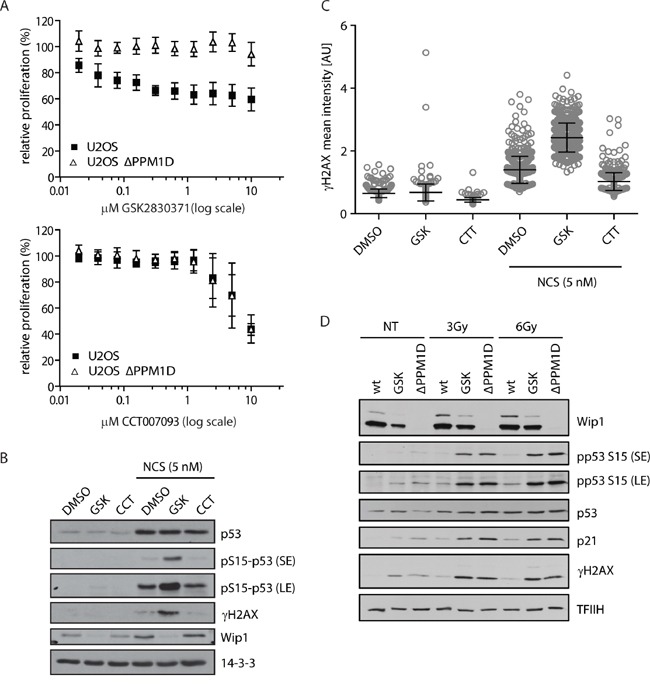
Validation of WIP1 inhibitors in U2OS-PPM1D-KO cells **A.** U2OS or U2OS-*PPM1D*-KO cells were treated with DMSO, CCT007093 or GSK2830371 at indicated doses and relative cell proliferation was measured after 7 days. Error bars represent SD. **B, C.** U2OS cells were treated with DMSO, CCT007093 (10 μM, CCT) or GSK2830371 (0.5 μM, GSK) and DNA damage was induced by 5 nM neocarzinostatin (NCS) for 5 h. Cells were analyzed by immunoblotting (B) or fixed and nuclear γH2AX intensity was determined by immunofluorescent staining and microscopy analysis **C.** Dots represent individual cells. Error bars represent SD. **D.** U2OS or U2OS-*PPM1D*-KO (ΔPPM1D) cells were treated with DMSO or GSK2830371 (0.5 μM), exposed to ionizing radiation (3 and 6 Gy) and analysed by immunoblotting using indicated antibodies. Short exposure (SE) or long exposure (LE) is shown.

To test the specificity of the novel WIP1 inhibitors in a cellular model, we generated U2OS-*PPM1D*-KO cells with the CRISPR-mediated knock-out of the *PPM1D* gene and determined the effect of CCT007093 or GSK2830371 compounds on cell growth (Figure 1A). Surprisingly, we have found that the effect of CCT007093 was not dependent on the presence of WIP1. In contrast, GSK2830371 showed a dose-dependent suppression of cell growth in parental U2OS but not in U2OS-*PPM1D*-KO cells. Next, we compared the ability of both compounds to potentiate a DNA damage-induced phosphorylation of two established substrates of WIP1 phosphatase, histone variant H2AX phosphorylated at Ser139 (referred to as γH2AX) and p53 phosphorylated at Ser15 (Figure 1B and 1C). We have not observed any significant differences in cells treated with DMSO and CCT007093 (10 μM) suggesting that CCT007093 does not block the activity of WIP1 in cells. In contrast, levels of γH2AX and pS15-p53 were increased in cells treated with GSK2830371 (0.5 μM) consistent with the expected inhibition of WIP1 activity. In accordance with a previous report we also observed a reduced level of WIP1 in the presence of an allosteric inhibitor GSK2830371 [63]. To further assess the efficiency of WIP1 inhibition, we compared responses to ionizing radiation in U2OS-*PPM1D*-KO cells and U2OS cells treated with GSK2830371 (Figure 1D). We found that treatment with GSK2830371 (0.5 μM) increased the phosphorylation of γH2AX and pS15-p53 and expression of p21 to comparable levels as the knock-out of *PPM1D* strongly indicating that GSK2830371 efficiently blocks WIP1 activity in cells.

**Figure 2:**
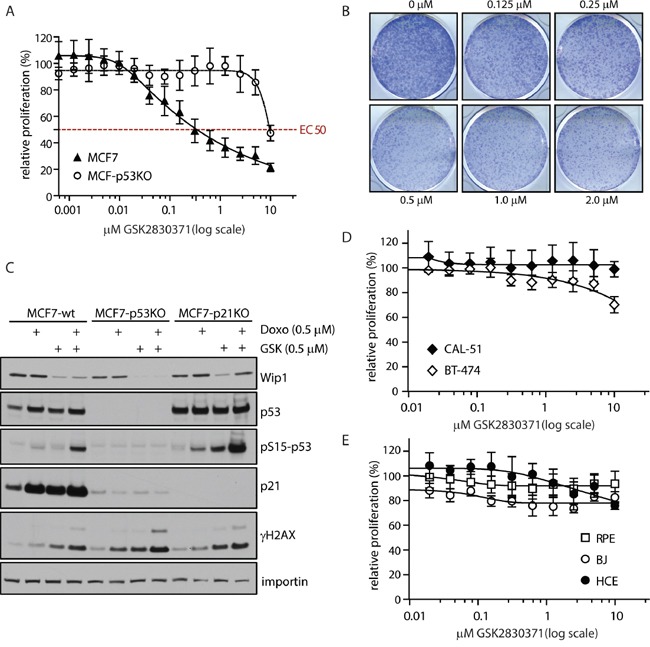
Inhibition of WIP1 impairs proliferation of cancer cells with amplified *PPM1D* **A.** MCF7 or MCF7-P53-KO cells were treated with indicated doses of GSK2830371 and relative cell proliferation was measured after 7 days. Error bars represent SD. **B.** MCF7 cells were treated with indicated doses of GSK2830371 and cell proliferation was determined by colony formation assay after 7 days. Representative image from three independent experiments is shown. **C.** MCF7, MCF7-P53-KO or MCF7-P21-KO cells were treated with DMSO, GSK2830371 (0.5 μM), doxorubicin (0.5 μM) or combination of both and cells were analyzed by immunoblotting after 24 h. **D.** BT-474 or CAL-51 cells were treated with indicated doses of GSK2830371 and relative cell proliferation was measured after 7 days. Error bars represent SD. **E.** BJ fibroblasts, hTERT-RPE1 cells or human colon epithelia cells (HCE) were treated with indicated doses of GSK2830371 and relative cell proliferation was measured after 7 days. Error bars represent SD.

Having established efficient concentration of GSK2830371 that specifically affects growth of U2OS cells, we continued with testing the sensitivity of breast cancer cells to GSK2830371. First, we tested the effect of WIP1 inhibition on cell proliferation in MCF7 cells that have massively amplified *PPM1D* locus at 17q22/q23 and harbouring wild-type p53 [31, 65]. Using cell proliferation and colony formation assays we observed dramatic reduction of cell growth after inhibition of WIP1 (Figure 2A and 2B). Reduction of cell proliferation by GSK2830371 showed EC50=0.3 μM in MCF7 cells which is in good agreement with a previous report [63]. In contrast, we have found that MCF7 cells with knocked-out *TP53* were less sensitive to GSK2830371 (Figure 2A and 2C). Similarly, we observed only a minor effect of GSK2830371 in BT-474 cells that contain amplification of the *PPM1D* but have inactivating mutation in *TP53* [65] (Figure 2D). Thus the effect of WIP1 inhibition on breast cancer cell proliferation depends on the intact p53 pathway as previously reported for haematological cancer cells [63]. Next we tested the sensitivity of CAL-51 breast cancer cells that contain a normal number of *PPM1D* alleles and wild type p53 (Figure 2D). We have found that CAL-51 cells were resistant to the treatment with GSK2830371 suggesting that cells with amplified *PPM1D* might be addicted to the high WIP1 activity whereas cells with normal levels of WIP1 can tolerate inhibition of WIP1 and proliferate also in the presence of GSK2830371. Finally, we tested the impact of GSK2830371 on proliferation of nontransformed cells. A dose of GSK2830371 that efficiently supressed growth of U2OS and MCF7 cells did not affect proliferation of BJ fibroblasts, hTERT-immortalized human retinal pigment epithelial cells (RPE) or SV40-immortalized human colon epithelia cells (HCE) indicating that inhibition of WIP1 is well tolerated by nontransformed cells (Figure 2E)

### WIP1 inhibition delays progression through G1 and G2 phases of the cell cycle

**Figure 3:**
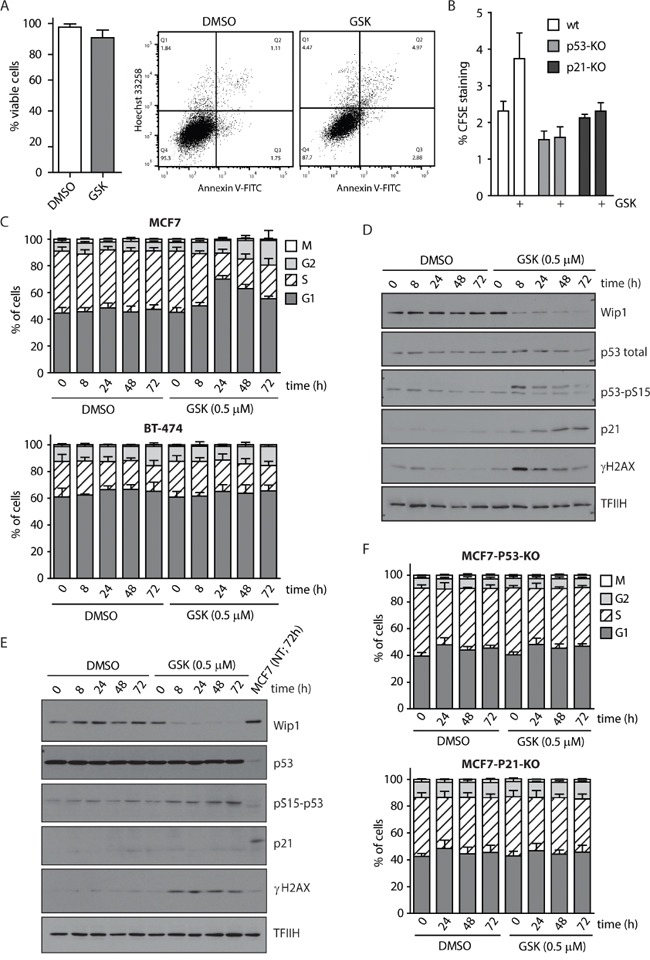
WIP1 inhibition leads to G1 and G2 phase accumulation in MCF7 cells **A.** MCF7 cells were treated with DMSO or GSK2830371 (0.5 μM) for 5 days and percentage of living cells (Hoechst/Annexin V negative) was determined by flow cytometry. Error bars represent SD. **B.** MCF7, MCF7-P53-KO or MCF7-P21-KO cells were incubated with CFSE and subsequently treated with DMSO or GSK2830371 (0.5 μM) for 3 days. Fluorescent signal of CFSE was measured by flow cytometry. Plotted is the CFSE signal relative to the signal measured at day 0. Error bars represent SD. **C.** MCF7 or BT-474 cells were treated with DMSO or GSK2830371 (0.5 μM) for indicated times, pulsed with BrdU before fixation and distribution of cell cycle phases was determined by flow cytometry. BrdU incorporation was used as a marker of replication and pS10-H3 as a marker of mitotic cells. Error bars represent SD. **D.** MCF7 cells were treated as in C and analyzed by immunoblotting. **E.** BT-474 cells were treated as in C and analyzed by immunoblotting. **F.** MCF7-P53-KO or MCF7-P21-KO cells were treated with DMSO or GSK2830371 (0.5 μM) for indicated times, pulsed with BrdU before fixation and distribution of cell cycle phases was determined as in C. Error bars represent SD.

Since we observed a strong reduction of the proliferating breast cancer cells population following WIP1 inhibition, we asked what the fate of the cells treated with GSK2830371 was. We found that GSK2830371 did not significantly affect the viability of MCF7 cells, suggesting that inhibition of WIP1 is not sufficient to induce cell death (Figure 3A). Instead we found that inhibition of WIP1 slowed down proliferation of MCF7 cells monitored by a dilution of CFSE dye in daughter cells (Figure 3B). The effect of GSK2830371 on the proliferation rate was fully dependent on p53 and p21 since we observed no differences in dilution of CFSE dye in MCF7-P53-KO or MCF7-P21-KO cells treated with WIP1 inhibitor (Figure 3B). Next we determined the effect of GSK2830371 on the cell cycle progression in MCF7 and BT-474 cells (Figure 3C). We have noted an accumulation of MCF7 cells in G1 phase 24 h after treatment with GSK2830371 (0.5 μM), whereas fraction of G2 cells was enriched in the later time points (48-72 h). This suggests that progression through G1 is slowed down in MCF7 cells early after addition of GSK2830371. Eventually cells progress through S phase to the G2 where they also progress more slowly compared to control cells. We did not observe any enrichment in the fraction of mitotic cells in the presence of GSK2830371 indicating that progression through mitosis was not affected by inhibition of WIP1 which is in good agreement with described degradation of WIP1 during prometaphase [66]. In contrast, no effect on the cell cycle progression was observed in BT-474, suggesting that observed extension of G1 and G2 phases depends on the ability to activate the p53 pathway (Figure 3C). Immunoblot analysis of MCF7 cells revealed that addition of GSK2830371 resulted in a rapid phosphorylation of p53 at Ser15 (Figure 3D). Two days after addition of GSK2830371, MCF7 cells showed increased levels of p21 which indicated a strong activation of the p53 pathway (Figure 3D). Consistent with no effect on the cell cycle progression and with the impaired p53 pathway, BT-474 cells did not show any induction of p21 levels after GSK2830371 administration (Figure 3E). Finally, we have found no effect on the cell cycle distribution in MCF7-P53-KO and MCF7-P21-KO cells treated with GSK2830371 further confirming that the effect of WIP1 inhibition on the progression through the cell cycle fully depends on the p53/p21 pathway (Figure 3F).

### WIP1 inhibition promotes DNA damage-induced checkpoint arrest

**Figure 4:**
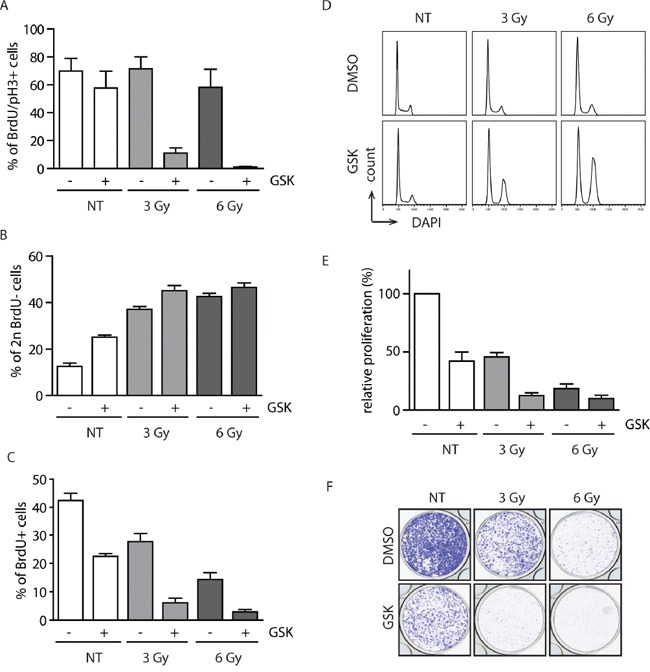
Inhibition of WIP1 potentiates the checkpoint through activation of the p53 pathway **A.** MCF7 cells were pulsed with BrdU, treated with DMSO or GSK2830371 (0.5 μM) and exposed to IR. Cells were incubated in the presence of nocodazole and collected after 20 h. Fraction of BrdU positive cells that progressed to mitosis (pH3 marker) was determined by flow cytometry. Error bars represent SD. **B.** MCF7 cells were treated as in A. Fraction of BrdU negative cells with 2n DNA content (corresponding to G1) was determined by flow cytometry 20 h after treatment. Error bars represent SD. **C, D.** MCF7 cells were treated with DMSO or GSK2830371 (0.5 μM), exposed to IR and BrdU incorporation (C) or cell cycle profile (D) was determined after 3 days. Error bars represent SD. **E.** MCF7 cells were treated with DMSO or GSK2830371 (0.5 μM), exposed to IR and cell proliferation was analyzed after 6 days. Error bars represent SD. **F.** MCF7 cells were treated as in E and cell proliferation was determined by colony formation assay after 6 days. Representative image from three independent experiments is shown.

We have previously shown that WIP1 is required for recovery from the DNA damage-induced G2 checkpoint [17]. Therefore, we tested the effect of GSK2830371 inhibitor on the ability of MCF7 cells to establish the G2 checkpoint. Whereas about 70 % of the control cells progressed to mitosis at 20 h after exposure to ionizing radiation, cells treated with GSK2830371 remained arrested in the G2 (Figure 4A). It has been reported that normal diploid RPE cells do not require WIP1 activity for recovery from the G1 checkpoint [18]. In the same time, C-terminally truncated WIP1 present in U2OS and HCT116 cells impairs activation of the G1 checkpoint [39]. To determine the contribution of the overexpressed WIP1 in suppression of the G1 checkpoint in MCF7 cells we compared fractions of cells remaining in G1 after exposure to ionizing radiation. Following exposure to a low dose of ionizing radiation (3 Gy, IR), MCF7 cells treated with GSK2830371 showed stronger accumulation in the G1 checkpoint compared to untreated cells (Figure 4B). To test how long these effects of WIP1 inhibition can persist we followed MCF7 cells for 3 to 6 days after irradiation and treatment with GSK2830371. We have found that cells with inhibited WIP1 did not incorporate BrdU three days after irradiation and that a substantial fraction of cells was arrested in the G2 checkpoint (Figure 4C and 4D). At 6 days after irradiation, we noted a dramatically reduced growth of cells exposed to a low dose (3 Gy) of IR and GSK2830371 (Figure 4E and 4F). Comparably smaller differences were observed after high dose of IR (6 Gy) when similar fractions of cells remained arrested regardless of the activity of WIP1 (Figure 4E and 4F).

### WIP1 inhibition sensitizes cells to genotoxic stress and to MDM2 inhibitor nutlin-3

**Figure 5:**
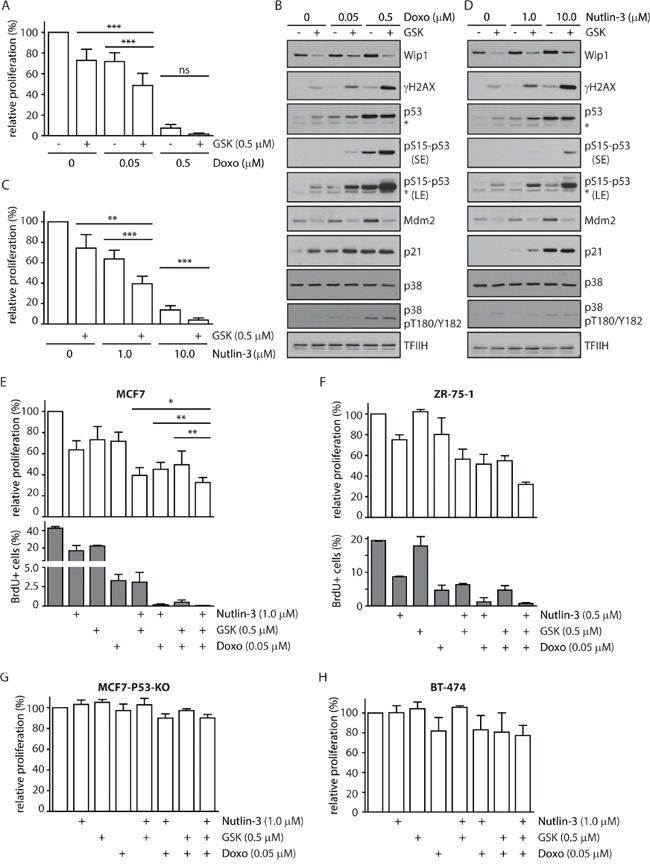
Inhibition of WIP1 increases sensitivity of cells to DNA damage and to nutlin-3 **A.** MCF7 cells were incubated with indicated doses of doxorubicin in combination with DMSO or GSK2830371 and relative fraction of proliferating cells was determined after 3 days. Error bars represent SD. **B.** MCF7 cells were incubated as in A and analysed by immunoblotting. Staining for TFIIH was used as loading control. Asterisk indicates an unspecific reactivity band. Short exposure (SE) or long exposure (LE) is shown. **C.** MCF7 cells were incubated with indicated doses of nutlin-3 in combination with DMSO or GSK2830371 and relative fraction of proliferating cells was determined after 3 days. Error bars represent SD. **D.** MCF7 cells were incubated with indicated doses of nutlin-3 and GSK2830371 for 1 day and analysed by immunoblotting. Staining for TFIIH was used as loading control. Asterisk indicates an unspecific reactivity band. Short exposure (SE) or long exposure (LE) is shown. **E.** MCF7 cells were incubated for 3 days with indicated doses of doxorubicin, nutlin-3 and GSK2830371 and fraction of proliferating cells was determined by cell survival assay (top) or by incorporation of BrdU (bottom). Error bars represent SD. **F.** ZR-75-1 cells were incubated for 6 days with indicated doses of doxorubicin, nutlin-3 and GSK2830371 and fraction of proliferating cells was determined by cell proliferation assay (top) or by incorporation of BrdU (bottom). Error bars represent SD. **G.** MCF7-P53-KO cells were incubated with indicated doses of doxorubicin, nutlin-3 and GSK2830371 and relative fraction of proliferating cells was determined after 3 days. Error bars represent SD. **H.** BT-474 cells were incubated with indicated doses of doxorubicin, nutlin-3 and GSK2830371 and relative fraction of proliferating cells was determined after 6 days. Error bars represent SD.

Since we observed potentiation of the IR-induced checkpoint arrest after inhibition of WIP1 we decided to test the combination of GSK2830371 with various chemotherapeutics causing genotoxic stress. High dose of doxorubicin (0.5 μM) strongly suppressed proliferation of MCF7 cells, which is consistent with extensive DNA damage caused by inhibition of topoisomerase II (Figure 4A). In contrast, low dose of doxorubicin (0.05 μM) caused only mild activation of p53 pathway and was relatively well tolerated in MCF7 cells (Figure 5A and 5B). Combined treatment with doxorubicin (0.05 μM) and GSK2830371 increased activation of the p53 pathway and significantly reduced proliferation of MCF7 cells (Figure 5A and 5B). Similar potentiation was observed also in combination of GSK2830371 and low doses of etoposide and bleomycin (data not shown). Together with the observed response to ionizing radiation (Figure 4E and 4F) this suggests that loss of WIP1 activity can potentiate DNA damage response to the low level of genotoxic stress whereas extensive DNA damage can trigger activation of this signaling cascade leading to a sustained growth arrest despite high expression levels of WIP1 present in MCF7 cells.

Transcriptional activity of the tumor suppressor p53 is regulated at multiple levels, including extensive phosphorylation in the transactivation and oligomerization domains and MDM2-dependent ubiquitination and degradation [67, 68]. Since inhibition of WIP1 increases phosphorylation of p53 at Ser15, we decided to test whether GSK2830371 could potentiate the effect of an MDM2 antagonist nutlin-3 that increases the total level of p53 [46]. As expected, treatment with high dose of nutlin-3 (10 μM) strongly suppressed cell proliferation of MCF7 cells (Figure 5C). Low dose of nutlin-3 (1 μM) showed an intermediate effect on cell proliferation of MCF7 cells that was further enhanced by simultaneous inhibition of WIP1 (Figure 5C). Consistent with an expected mode of action, we observed increased levels of total p53 after treatment with nutlin-3, increased phosphorylation of p53 at Ser15 after treatment with GSK2830371 and both effects after combined treatment with both inhibitors (Figure 5D). Efficient inhibition of WIP1 is documented by increased basal phosphorylation of γH2AX which is an established substrate of WIP1 and also by decreased levels of MDM2 which is destabilized in the absence of WIP1 activity (Figure 5B and 5D) [14, 20, 22]. Although inhibition of WIP1 slightly increased the basal phosphorylation of p38 at Thr180/Tyr182 (established substrate of WIP1), we did not observe any further increase of p38 activity in combination of GSK2830371 with doxorubicin or nutlin (Figure 5B and 5D). This suggests that p38 does not potentiate the cytotoxic effect of WIP1 and WIP1 impacts on p53 independently on the p38 pathway.

Finally, we tested the potentiation of the cytostatic effect by combining the GSK2830371 with low doses of nutlin-3 and doxorubicin. We found that this triple combination further decreased cell proliferation of MCF7 cells compared to treatments with individual drugs or with the double inhibitor combinations (Figure 5E). Triple combination of GSK2830371, nutlin-3 and doxorubicin also potentiated the cytostatic effect in ZR-75-1 cells that contain amplification of the *PPM1D* locus and harbour wild-type p53 (Figure 5F). In contrast no potentiation was observed in BT-474 and MCF7-P53-KO cells strongly indicating that status of p53 plays a key role in determining the cell sensitivity to WIP1 inhibition (Figure 5G and 5H).

### Inhibition of WIP1 potentiates activation of p53 pathway

**Figure 6:**
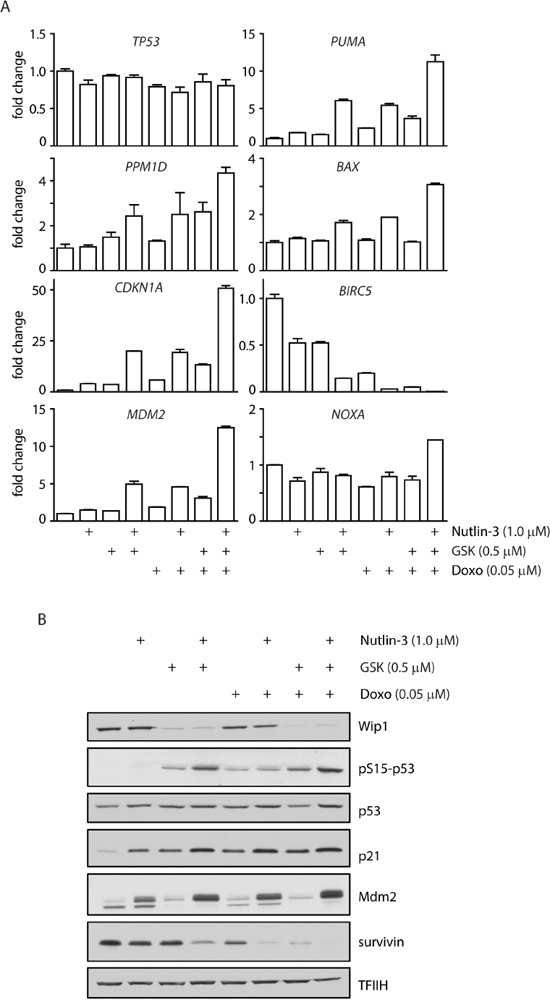
Inhibition of WIP1 increases transcription of p53 target genes **A.** MCF7 cells were incubated for 3 days with indicated doses of doxorubicin, nutlin-3 and GSK2830371 and expression of indicated genes was determined by qRT-PCR. Levels are presented as the ratio of mRNA to GAPDH mRNA and are normalized to untreated cells. Error bars correspond to SEM. **B.** MCF7 cells were incubated as in A and expression of selected proteins was analysed by immunoblotting.

To quantify activation of the p53 pathway after treatment of MCF7 cells with combination of WIP1 inhibitor and chemotherapeutics we analyzed the expression profiles of selected established p53 target genes. As expected, expression of *CDKN1A* increased 3-5 fold after treatment with GSK2830371, nutlin-3 or doxorubicin administered individually (Figure 6A). Double combination of GSK2830371 with nutlin-3 or doxorubicin resulted in approximately 20 fold increase in *CDKN1* expression. The highest induction of *CDKN1A* expression (about 50 fold) was observed after triple combination of GSK2830371, nutlin-3 and doxorubicin. Similarly, expression of p53 up-regulated modulator of apoptosis (*PUMA*) or pro-apoptotic regulator *BAX* showed the strongest induction after triple combination of GSK2830371, nutlin-3 and doxorubicin. In contrast, we did not observe any significant change in expression of an apoptosis-promoting gene *NOXA*. Inversely, we observed a strongly reduced expression of *BIRC5* (coding for survivin), an anti-apoptotic gene that was reported to be suppressed in a p53-dependent manner [69, 70]. In addition, we have found strongly increased expression of *PPM1D* and *MDM2* after triple combination of GSK2830371, nutlin-3 and doxorubicin, which is consistent with the described transcriptional regulation of both genes by p53. Although expression of *PPM1D* mRNA was increased after triple combination of the drugs, protein levels of WIP1 were decreased (Figure 6B) due to the destabilization of WIP1 caused by binding of GSK2830371 to its catalytic domain [63]. After 3 days of GSK2830371 treatment we did not observe increased total levels of p53; however p53 was heavily phosphorylated at Ser15 known to stimulate its transcriptional activity [11].

### Inhibition of WIP1 promotes induction of senescence and apoptosis

**Figure 7:**
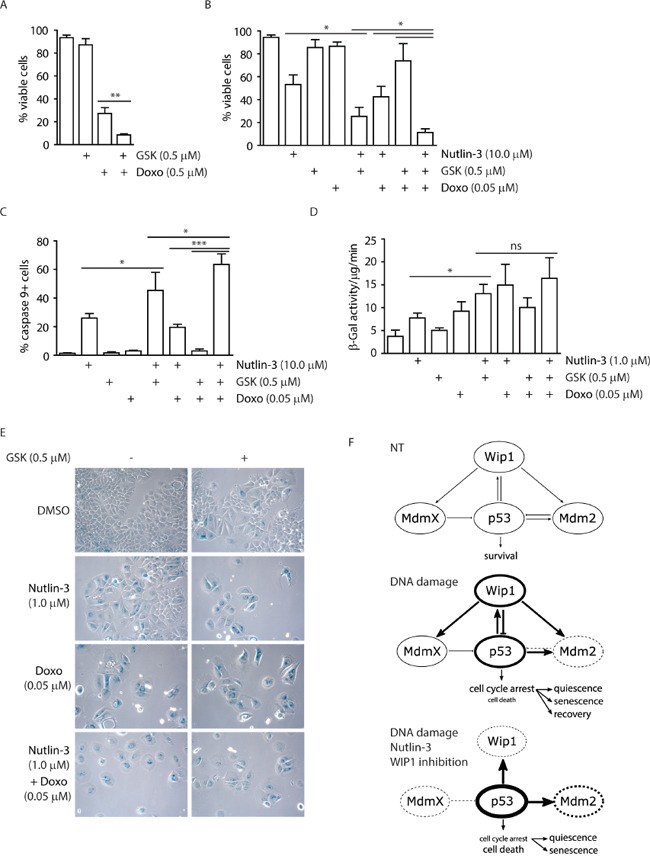
Inhibition of WIP1 potentiates induction of senescence or apoptosis **A.** MCF7 cells were incubated with GSK2830371 (0.5 μM) and doxorubicin (0.5 μM) for 3 days and fraction of viable cells (Hoechst/Annexin V negative) was determined by flow cytometry. Error bars represent SD. **B.** MCF7 cells were incubated with indicated combinations of GSK2830371 (0.5 μM), nutlin-3 (10.0 μM) and doxorubicin (0.05 μM) for 3 days and fraction of viable cells (Hoechst/Annexin V negative) was determined by flow cytometry. Error bars represent SD. **C.** MCF7 cells were treated as in (B) and fraction of cells with active caspase 9 was determined by flow cytometry. Error bars represent SD. **D.** MCF7 cells were incubated with indicated combinations of GSK2830371, nutlin-3 (1.0 μM) and doxorubicin (0.05 μM) for 7 days. Activity of SA-β-galactosidase was measured in cell extracts using fluorimetric assay. Error bars represent SD. **E.** MCF7 cells were incubated as in D and SA-β-galactosidase activity was evaluated by light microscopy. **F.** Model for outcomes of treatment with p53/mdm2/Wip1 pathway modulators. Under non-treated conditions, p53 activity is tightly controlled by MDM2 and MDMX. Upon mild DNA damage, MDM2 is inhibited and destabilized leading to stabilization of p53 that in turn leads to increased transcription of its targets including WIP1 phosphatase. Subsequently WIP1 inactivates p53 pathway by direct dephosphorylation of p53 Ser15 and through activation of MDM2 and possibly also MDMX by their dephosphorylation. When MDM2-p53 interaction inhibitor nutlin-3 and WIP1 inhibitor are combined with DNA damage, MDM2 cannot ubiquitinate and thus degrade p53 and WIP1 cannot oppose activation of p53. This leads to further increase of p53 protein levels and its phosphorylation at Ser15 and results mainly in cell death. Thickness of the circle lines represents protein levels; dashed lines mean inhibition of the protein activity.

Since the expression profiling showed induction of the checkpoint and pro-apoptotic genes, we asked what the fate of cells treated with WIP1 inhibitor alone or in combination with other chemotherapeutics was. Although, cell proliferation was suppressed in MCF7 cells treated with GSK2830371, we observed only mild reduction in the fraction of viable cells compared to the control cells (Figure 3A). In contrast, GSK2830371 significantly decreased viability of MCF7 cells when administered concomitantly with a high dose of doxorubicin (0.5 μM) while having only mild effect when administered together with low dose of doxorubicin (0.05 μM) (Figure 7A, 7B). Similarly, GSK2830371 decreased viability of MCF7 cells treated with a high dose of nutlin-3 (10.0 μM) (Figure 7B). Consistent with a previous report, nutlin-3 increased sensitivity of cells to the low dose of doxorubicin (0.05 μM) [71]. Moreover, we have observed that GSK2830371 further increased the sensitivity of MCF7 cells to a combined treatment with nutlin-3 and doxorubicin (Figure 7B). This suggests that inhibition of WIP1 can potentiate cytotoxic effects of doxorubicin and the MDM2 antagonist nutlin-3. In addition, we observed induction of caspase 9 activity after combined treatment with GSK2830371, nutlin-3 and doxorubicin which is consistent with activation of an intrinsic apoptotic pathway (Figure 7C) [72].

Whereas combination of the high dose of nutlin-3 and GSK2830371 efficiently induced cell death, most cells survived treatment with the low dose of nutlin-3. Since these cells did not incorporate BrdU (Figure 5E), we hypothesized that they corresponded to the population of cells permanently withdrawn from the cell cycle. Indeed, MCF7 cells treated with GSK2830371 and 1.0 μM nutlin-3 exhibited flattened and enlarged morphology; and showed induction of β-galactosidase activity, both established markers of cellular senescence (Figure 7D and 7E) [73].

In summary, we have validated GSK2830371 as potent and specific inhibitor of WIP1 phosphatase. Our data suggest that mild activation of p53 pathway caused by a partial stabilization (through low levels of nutlin-3) or phosphorylation of p53 (through inhibition of WIP1) is sufficient to slow down proliferation and eventually promotes cellular senescence. Conversely, full activation of p53 pathway achieved by combined effects of genotoxic stress with inhibition of two negative regulators of p53, MDM2 and WIP1 can potentiate cell death in breast cancer cells (Figure 7F).

## DISCUSSION

Taking advantage of the U2OS cells with knocked-out *PPM1D*, we compared effects of the two commercially available inhibitors of WIP1 phosphatase in a cellular model. Data presented here and also by others strongly suggest that CCT007093 compound suppresses the cell growth independently of WIP1 inhibition [59]. It is possible that CCT007093 stimulates the p38 pathway as originally reported, however caution should be taken when interpreting these effects as a result of WIP1 inhibition. In contrast, our cellular model confirmed the specificity of the novel allosteric inhibitor GSK2830371 that interfered with dephosphorylation of γH2AX (an established substrate of WIP1) and suppressed cell growth in a WIP1-dependent manner. Notably, an impact of GSK2830371 on activation of the DNA damage response pathway was comparable to that of the *PPM1D* knock out indicating that GSK2830371 can efficiently inhibit WIP1 in cells.

We have found that GSK2830371 administered at doses that specifically block WIP1 activity does not affect proliferation of nontransformed cells but impairs proliferation of breast cancer cells with amplified *PPM1D*. MCF7 cells treated with GSK2830371 accumulate over time in the G2 phase of the cell cycle. This observation is in good agreement with the higher ratio of the G2 cells reported in the population of PPM1D−/− MEFs compared to the wild type MEFs and also with the increased expression level of WIP1 during the G2 in human cells [66, 74]. Analyzis of the MCF7-P53-KO and MCF7-P21-KO cells has shown that this effect of WIP1 on the cell cycle progression is mediated by the p53/p21 pathway. Level of p21 present during G2 was recently identified as an important factor that determines the fate of proliferating cells [75, 76]. Low level of p21 in G2 allows immediate building up of the CDK2 activity following mitotic exit and results in continuous proliferation. In contrast, cells with high level of p21 during G2 remain temporarily arrested in a quiescence after completing cell division and do not proliferate unless stimulated with excessive dose of growth factors [75]. It is plausible that these cells eventually become senescent after long period of sustained p21-dependent inhibition of cyclin dependent kinases. It appears that cells progressing through G2 phase are very sensitive to activation of the p53/p21 pathway. Indeed, short activation of p53 during G2 triggered nuclear retention and subsequent degradation of Cyclin B1 and was sufficient to induce a permanent withdrawal from the cell cycle [77, 78]. Here we have shown that inhibition of WIP1 potentiates an effect of a low dose of nutlin-3 resulting in increased induction of senescence in breast cancer cells.

Although GSK2830371 efficiently suppressed growth of breast cancer cells with amplified *PPM1D* and wild type *TP53*, it did not affect viability of MCF7 cells suggesting that inhibition of WIP1 alone may not be sufficient to eradicate tumor cells. On the other hand, we have found that inhibition of WIP1 by GSK2830371 potentiated doxorubicin-induced cell death in breast cancer cells. This data is consistent with previously reported high sensitivity of Wip1-depleted MCF7 cells to doxorubicin [79]. Similar potentiation of the cytotoxic effect of doxorubicin by WIP1 inhibition has recently been reported in neuroblastoma cells and in a colorectal carcinoma cells with a C-terminally truncated *PPM1D* [61, 64]. In addition, we have found that inhibition of WIP1 potentiated cell death induced by nutlin-3. Synergistic effect of nutlin-3 and doxorubicin has been reported in B-cell leukemia and in breast cancer cells [71, 80]. Here we show that combination of GSK2830371 with doxorubicin and nutlin-3 further increased activation of the p53 pathway and resulted in massive cell death. Clinical outcome of doxorubicin therapy can be impaired by induction of senescence in breast cancer cells with wild-type p53 [81, 82]. Strong induction of p53 function by concomitant inhibition of WIP1 and/or MDM2 could increase the fraction of cells eliminated by cell death and thus could improve the response to doxorubicin. In addition, therapeutic effect of doxorubicin is limited by a cumulative, dose-related cardiotoxicity [83]. Possible reduction of the doxorubicin dose administered in combination with WIP1 inhibitor could be beneficial for breast cancer patients by decreasing undesired side effects of chemotherapy.

WIP1 has been reported to directly target several proteins implicated in apoptosis (including BAX and RUNX2) in p53 negative cells [84–86]. However, suppression of cell growth and induction of cell death by WIP1 depletion or inhibition fully depends on the p53 pathway. In addition, inhibition of WIP1 efficiently affects growth of cells with amplified or truncated *PPM1D* whereas little effect is observed in cells with normal levels of WIP1. This suggests that determination of the status of *TP53* and *PPM1D* in the tumors will be important for predicting the therapeutical outcome of WIP1 inhibitors. Further research is needed to identify additional factors determining the sensitivity of cancer cells to WIP1 inhibitors. Response of cancer cells to nutlin-3 depends on the level of MDM2 and is commonly impaired by overexpression of MDMX [71, 87, 88]. Since GSK2830371 potentiates the cytotoxic effect of nutlin-3, we hypothesize that *MDMX* overexpressing tumors might be attractive candidates for testing the sensitivity to WIP1 inhibition.

## MATERIALS AND METHODS

### Cell lines

Human osteosarcoma U2OS and breast cancer MCF7 cells were generous gifts from Dr. Medema (NKI, Amsterdam), BT474 from Dr. Truksa (IBT, Prague), CAL-51 and BJ fibroblasts (population doubling 40-50) from Dr. Bartek (IMG, Prague). ZR-75-1 cells were obtained from European Collection of Cell Cultures, hTERT-RPE1 from ATCC and human SV40-immortalized colon epithelia HCE cells from Applied Biological Materials (ABM, #T0570). Cells were grown at 37°C and 5% CO2 in DMEM, RPMI (ZR-75-1 and BT-474) or Prigrow III media (HCE cells) supplemented with 6-10% FBS (Gibco), penicillin (100 U/ml), and streptomycin (0.1 mg/ml). All cell lines were regularly checked for absence of mycoplasma infection using MycoAlert Plus reagent (Lonza). To knock-out *TP53* or *CDKN1A* gene, MCF7 cells were transfected with a combination (1:1) of p53 CRISPR/Cas9 KO Plasmid (Santa Cruz, sc-416469) or p21 CRISPR/Cas9 KO Plasmid (sc-400013) and corresponding HDR Plasmids and stable clones were selected by puromycin (10 μg/ml). Integration of the HDR cassette to genomic loci was confirmed by sequencing and loss of protein expression by immunoblotting. To generate *PPM1D* knock-out cells, U2OS cells were transfected with a CAS9-2A-GFP plasmid expressing the gRNA corresponding to the tgagcgtcttctccgaccaggg sequence in exon 1 of the human *PPM1D* (Sigma). Individual GFP positive clones were expanded and loss of WIP1 expression was determined by immunoblotting. Transfection of plasmid DNA was performed using Lipofectamine LTX according to recommendations of manufacturer (Life Technologies). Where indicated, cells grown on culture plates were exposed to ionizing radiation generated by X-ray instrument T-200 (16.5 Gy/min, Wolf-Medizintechnik).

### Antibodies and chemicals

The following antibodies were used: WIP1 (sc-130655), p53 (sc-6243), TFIIH (sc-293), importin (sc-137016), p21 (sc-397) from Santa Cruz; pSer15-p53 (#9284), γH2AX (#9718), p38 MAPK Thr180/Tyr182 (#9216S) and p38 MAPK (#9212) from Cell Signaling Technology); γH2AX (05-636, Millipore); MDM2 (Calbiochem); Alexa Fluor-labelled secondary antibodies (Life Technologies); anti-BrdU FITC-conjugated antibody (#347583, BD Biosciences) and anti-pSer10-H3 antibody (Upstate). Doxorubicin hydrochloride (Sigma), GSK2830371 and nutlin-3 (both MedChem Express) were diluted in DMSO and used at indicated doses. Resazurin, neocarzinostatin (NCS) and carboxyfluorescein diacetate succinimidyl ester (CFSE) were purchased from Sigma.

### Cell proliferation assay

MCF7 or BT-474 cells were seeded into 96-well plates at 2×10^3^ or 0.5×10^3^ cells/well, treated with a compound dilution series and analyzed after 3 or 7 days, respectively. CAL-51, RPE, HCE, BJ or ZR-75-1 cells were seeded into 96 well plates at 0.02-2×10^4^ cells/well and grown for 7 days. Resazurin (30 μg/mL) was added to growth media and fluorescence signal (excitation wavelength 560 nm, emission wavelength 590 nm) was measured after 1 to 5 h using EnVision plate reader (PerkinElmer).

Alternatively, rate of cell proliferation was determined using CFSE Cell Proliferation assay as previously described [89]. Cells were stained with 50 μM CFSE in complete media for 15 min in 37°C, washed with complete media and seeded to 12-well plate at 2.5×10^4^ cells/well. Where indicated, GSK2830371 (0.5 μM) was added to the media. Cells were harvested and fixed by 4% paraformaldehyde 3 days after treatment. Percentage of the remaining CFSE staining compared to the cells harvested immediately after staining was determined by flow cytometry.

### Clonogenic assay

Cells were seeded in 6-well plates at 2×10^4^ cells/well. Cells were treated with a compound dilution series on day 1. After 6-7 days, cells were washed with PBS, fixed by 70 % ethanol for 15 min and stained with crystal violet dye.

### Cell cycle assay

Cells were grown for indicated times in the presence of DMSO or GSK2830371 (0.5 μM), pulsed with BrdU (10 μM for 30 min; Sigma), harvested by trypsinization and fixed in ice-cold 70 % ethanol. Following the protocol from manufacturer, cells were stained with anti-BrdU-FITC (replication marker, BD Biosciences), anti-pSer10H3 (mitotic marker) and DAPI and analyzed by flow cytometry using LSRII (BD Biosciences) and FlowJo software (FlowJo).

### Checkpoint analysis

Evaluation of the cell cycle checkpoints was performed as described previously with minor modifications [39]. Cells were pulsed with BrdU (10 μM for 30 min) and treated with GSK2830371 (0.5 μM) or DMSO before irradiation with 3 or 6 Gy and were grown for further 20 h in the presence of nocodazole (250 ng/ml). Cells were processed as mentioned above and analyzed by flow cytometry. BrdU-positive cells were assayed for progression through the G2 phase to mitosis (4n DNA content, pH3+). BrdU-negative cells with 2n content were used for quantification of cells arrested in G1 checkpoint.

### Cell viability assay

MCF7 cells were seeded into 12-well plates at 2×10^4^ cells/well, treated with Nutlin-3 (10 μM or 1 μM), GSK2830371 (0.5 μM) and doxorubicin (0.05 μM or 0.05 μM) and grown for 3 days. Cells were trypsinized, washed with PBS and incubated with FITC-conjugated Annexin V (BD Biosciences) and Hoechst-33258 for 15 minutes. Fraction of living cells was determined as Annexin V negative and Hoechst negative population analyzed by flow cytometry.

### β-galactosidase assay

Senescence-associated β-galactosidase activity was quantified in cell extracts as previously described [90]. Briefly, MCF7 cells were seeded into 6 cm plates at 0.5×10^5^ cells/plate and grown in media supplemented with indicated combinations of nutlin-3 (1 μM), GSK2830371 (0.5 μM) and doxorubicin (0.05 μM) for 7 days. Cells were washed in PBS, collected to ice cold lysis buffer (5 mM CHAPS, 40 mM citric acid, 40 mM sodium phosphate, 0.5 μM benzamidine and 0.25 mM PMSF, pH 6.0), vortexed and centrifuged for 5 min at 12,000g. Cell extract was mixed 1:1 with 2x reaction buffer supplemented with 4-MUG (1.7 mM, Sigma) and MgCl2 (4 mM) and incubated at 37°C for 0.5 – 4 hours. Reaction was stopped by addition of sodium carbonate (400 mM) and fluorescence signal was measured at excitation wavelength 360 nm and emission wavelength 465 nm using EnVision plate reader. β-galactosidase activity was determined as the rate of 4-MUG conversion to the fluorescent 4-MU and normalized to the protein concentration measured by BCA assay. Alternatively, cells were grown on coverslips, fixed by 0.2 % glutaraldehyde 7 days after treatment with indicated combinations of nutlin-3 (1 μM), GSK2830371 (0.5 μM) and doxorubicin (0.05 μM) and β-galactosidase activity was determined by colorimetric staining as previously [73].

### Caspase assay

Activity of caspase-9 was measured using SR-FLICA Caspase-9 assay according to manufacturer protocol (Immunochemistry Technologies). Briefly, cells were seeded to 12-well plates, treated as indicated, harvested by trypsinization after 48 h and re-suspended in complete media containing SR-FLICA caspase-9 and incubated 1 h at 37°C. After incubation, cells were washed with Apoptosis wash buffer for 10 min at 37°C. Percentage of cells positive for caspase-9 activity was determined by flow cytometry.

### Immunofluorescence microscopy

U2OS cells grown on coverslips were treated with DMSO, CCT007093 or GSK2830371 for 1 h and DNA damage was induced by neocarzinostatin for 5 h. Cells were fixed by 4 % formaldehyde (10 min at RT), permeabilized by ice-cold methanol and stained with antibody against γH2AX and with DAPI. Average nuclear intensity of γH2AX signal was quantified using Scan^R high-content screening station as described previously [66].

### Quantitative real-time PCR (qPCR)

Total RNA was isolated using RNeasy mini kit (Qiagen). cDNA was synthetized using 0.5 μg RNA, random hexamer, and RevertAid H Minus Reverse Transcriptase (Thermo Scientific). RT-qPCR was performed using LightCycler 480 SYBR Green I Master mix; Light Cycler LC480 (Roche) and following cycle conditions: initial denaturation 95°C for 7 min, followed by 45 cycles of denaturation 95°C for 15 s, annealing 60°C for 15s and extension 72°C for 15s. A melting curve analysis was used to confirm the specificity of amplification, and Ct values were determined using LigtCycler480 software. All data are presented as the ratio of the tested mRNA to *GAPDH* mRNA. Primers are listed in the table.

**Table:** 

Gene	Forward sequence	Reverse sequence
*PPM1D*	CTGAACCTGACTGACAGCCC	CTTGGCCATGGATCCTCCTC
*BIRC5*	CTGCCTGGTCCCAGAGTG	GTGGCACCAGGGAATAAACC
*MDM2*	TCGACCTAAAAATGGTTGCAT	GGCAGGGCTTATTCCTTTTC
*PUMA*	TCTCGGTGCTCCTTCACTCT	ACGTTTGGCTCATTTGCTCT
*BAX*	GCTGGACATTGGACTTCCTC	GTCTTGGATCCAGCCCAAC
*CDKN1A*	GGCGGCAGACCAGCATGACA	CCTCGCGCTTCCAGGACTGC
*TP53*	CAGCACATGACGGAGGTTGT	TCATCCAAATACTCCACACGC
*NOXA*	GCTGGGGAGAAACAGTTCAG	AATGTGCTGAGTTGGCACTG

### Statistical analysis

Statistical analysis was performed in GraphPad Prism 5.04 software. Statistical significance was determined from at least three independent experiments using a paired two-tailed T-test (* corresponds to p-value < 0.05; ** p-value < 0.005; *** p-value < 0.0005). Error bars indicate standard deviations. EC50 was calculated using Richard's five-parameter dose-response curve for non-linear fitting analysis.
